# Two tales of platform regimes in China’s food-delivery platform economy

**DOI:** 10.1186/s40711-022-00170-5

**Published:** 2022-08-01

**Authors:** Haitao Wei, Luyang Zhang, Peipei Deng, Guohui Li

**Affiliations:** School of Sociology, Central China Normal University, Wuhan, China

**Keywords:** Platform despotism, Food-delivery platform, Couriers, Platform regime, China

## Abstract

This article brings the often-overlooked concept of the labor regime back to the study of China’s food-delivery platform workers. Two tales of platform regimes emerge: individualized platform despotism and bureaucratized platform despotism, which apply to crowdsourcing couriers and dedicated delivery couriers, respectively. This study compares these two types of platform regimes in terms of their institutional foundation and labor organization. Despite different institutional arrangements and labor organization, both types of food-delivery couriers belong to a despotic platform regime revealing workers’ subordination to the platform. In conclusion, it discusses the implications and limitations of this study.

## Introduction

Over the last decade, rapidly expanding information communication technologies (ICTs), big data, and cloud computing have enhanced digitally-enabled platforms, which has overwhelmingly transformed the worldwide economy (De Stefano 2016; Kenney and Zysman 2016; Srnicek 2017). Different types of platform enterprises have emerged across the world. In 2017, the combined value of the platform companies with a market capitalization of more than $100 million was estimated at more than $7 trillion—67% higher than in 2015 (United Nations 2019: xvii). China is one of the centers of this wave, with more than 75 million people working on digital platforms,^1^ which creates a large-scale digital working class (Qiu 2018). This article focuses on the food-delivery industry and the platform workers within it.

The scale of the food-delivery industry in China has gone through tremendous growth in the past few years. It was estimated that the scale of the online food-delivery industry was 664.6 billion RMB in 2020, 15% higher than in the previous year.^2^ The COVID-19 pandemic further accelerated this growing process. Accompanying this industrial growth, many job seekers enter this industry and choose to become food-delivery couriers. There are two dominant food-delivery platforms in China, Meituan and Ele.me, which account for more than 90% of couriers. For example, in 2020, the number of registered couriers in Ele.me was 3 million, while the size in Meituan was close to 4 million.^3^ Based on the survey reports released by the two platforms, 80% of platform workers come from rural areas concentrated in Henan, Anhui, and Sichuan provinces. The workers are male-dominated (92%), and the average age is 30.^4^ In the report of the Ele.me platform, more than 80% of workers chose the work for its flexibility, and most of them were factory workers before joining the food-delivery industry.^5^

There are two types of food-delivery platform workers in China: dedicated delivery couriers (*Zhuansong* in Chinese) and crowdsourcing couriers (*Zhongbao* in Chinese). The dedicated delivery couriers are manufactured by the platforms to maintain a disposable and reliable labor force, while the crowdsourcing couriers are made to keep a flexible labor force (Lei 2021). Both Meituan and Ele.me operate the *Zhuansong* and *Zhongbao* service platforms. The workers themselves decide whether they want to be *Zhuansong* or *Zhongbao* couriers. These two companies subcontract the food-delivery business to other companies, which set up and operate service stations in specific areas to provide food-delivery service. The franchisees establish district managers and station supervisors to manage the couriers. The service stations are responsible for recruiting *Zhuansong* couriers and managing the labor force with the technological system provided by the platform. *Zhongbao* couriers simply register via the app, log in to take orders, and compete for orders independently; that is, apart from the platform, they are not supervised by any other organization. *Zhongbao* workers can decide when to log into the app to start working. Due to their differences, our article aims to compare these two types of platform workers in terms of their working conditions and the institutional environments that shape them.

Many sociological studies have explored the working conditions of platform workers, especially through the lens of labor process theory. However, to our best knowledge, scant research has examined the configuration of labor regimes among platform workers, which comprehensively considers multifaceted dimensions of workers’ control and management. This study aims to bring the concept of the labor regime back to the study of platform workers. The concerning question here is: how is the labor regime of food-delivery couriers configured? With Burawoy’s conceptualization of the production regime, we explore and compare similarities and differences between dedicated delivery couriers and crowdsourcing couriers in China.

Theoretically, this article proposes the concept of a platform regime referring to the specific labor regime in the platform economy. The platform regime delineates the working conditions of platform workers and the institutional apparatuses shaping the labor relations between the platform and workers. Empirically, this study presents how institutional arrangements create a despotic platform regime among food-delivery couriers in China. Furthermore, it also shows two different subtypes of this despotic platform regime. The first is an individualized platform despotism characterized by individual and sole interactions between workers and the platform. The crowdsourcing couriers (*Zhongbao* workers) apply to this type of platform regime, in which the platform algorithm plays a dominant role in its labor organization. The second is a bureaucratized platform despotism characterized by workers’ double subordination to organizational and algorithmic control and management simultaneously. The dedicated delivery couriers (*Zhuansong* workers) apply to the regime in which the hierarchical organization and authoritative personnel play significant roles in labor organization and control. We compare these two types of platform despotism regarding institutional foundation and labor control.

In what follows, we first revisit the literature on platform workers revolving around their labor process and build a basic analytic framework to compare the two types of food-delivery couriers. Then, we present the two types of platform regimes with empirical data. In conclusion, we summarize our findings and discuss the implications of our study.

## Labor process of platform workers

The platform economy creates new jobs and transforms old jobs, ranging from transportation, logistics, and the food industry to online gig work or crowd work (Prassl and Risak 2016; Howcroft and Bergvall-Kreborn 2019; Wood et al. 2019). The platformization of the service industry also creates different types of workers depending on the platform for obtaining income. While legal scholars are mainly interested in exploring the ambivalent legal role of platform workers because of the ambivalence of existing legal regulations (Aloisi 2016; Holloway 2016; Malin 2018), sociological studies on platform workers largely focus on how the labor process is organized or the dynamics between control and resistance among platform workers.

In the platform economy, the internet platform can transcend the limitation of stable temporality and spatiality to match the supply and need of the labor force in a timely manner, which creates the digitalization of workers’ labor processes (Stanford 2017; Wood et al. 2019). Sociologists have comprehensively recorded the distinctive features of the labor organization of platform work via the assistance of digitally enabled infrastructure and new advances in technology, characterized by "fissuring of the workplace" (Weil 2014), "digital Taylorism" (Cherry 2016), and a trend of the disembeddedness of labor (Wood et al. 2019). Saliently, data become a valuable resource for platforms, enabling them to track, record, and codify the data that different users produce so the platforms can utilize the data to make profits (Doorn and Badger 2020). Based on the enormous amount of data they collect, platforms can direct, monitor, and control platform workers through algorithms such as “algorithmic management” (Kyung et al. 2015), “technology-normative control mode” (Gandini 2019), “technology-mediated control” (Wiener et al., 2020) or “digital control” (Chen 2020).

The main features of this control mode centered on "algorithms" can be summarized as follows. First, the platform app becomes the center of the labor organization for both the platform workers and the platform enterprise (Gandini 2019; Veen et al. 2020). Through the users’ clickwrap agreement, users’ page, the guidance of users’ labor process, and evaluation of users’ performance, the platform has absolute authority and becomes a nonnegotiable ‘employer’ (Moore et al. 2017; Srnicek 2017:47). Second, multiple mechanisms are designed and applied by the platform to reinforce its management, control, and supervision of platform workers. For example, the piece-rate incentive system based on algorithmic management that can flexibly adjust the wage structure according to the changing market environment encourages workers to put more effort into their work (Rosenblat and Stark 2016; Gandini 2019). Furthermore, the platform constantly and efficiently collects and records workers’ activity data during the labor process, which benefits the platform optimization of their algorithms, eventually contributing to the platform’s stringent control and precise projection of platform workers’ activities (Chen 2020). Finally, customers are introduced by the platform into the workers’ management and control process via the algorithmic evaluation system (Veen et al. 2020). Customers are given the power to evaluate workers’ performance with the rating mechanism, which accelerates the interaction and conflict between platform workers and customers, leaving the platform more as a moderator between them (Rosenblat and Stark 2016; Kirven 2018).

While platform workers are subject to the platform monopoly and its centralized algorithmic management, platform workers could recognize those algorithms and fight against the rise of the platform (Chen 2018; Sun 2019; Sun and Chen 2021) because of a “structured antagonism” between the platform and labor (Wood and Vili 2019). Labor scholars also record platform workers’ resistance during and beyond the labor process. During the labor process, platform workers can identify the technical loopholes of the algorithms and use them to realize their interests (Jarrahi and Sutherland, 2018; Sun and Chen, 2021). The practices of workers’ everyday resistance include sharing accounts, buying reviews on platforms, negotiating working hours and wages, and creating multiple accounts (Anwar and Graham 2019; Wood et al. 2019). Due to the fragmentation of the labor process and atomization of platform workers, it is assumed that it is difficult for platform workers to initiate collective action (Heiland 2020). However, platform workers could still overcome the obstacles and collectively fight against the platform through workers’ online communicative networks (Lehdonvirta 2016; Tassinari and Maccarrone 2020).

Sociological studies on platform workers’ labor process show us how workers are subject to the platform and workers’ responses to platform algorithmic management. However, there are two weaknesses in the literature. First, while existing studies have greatly added to our knowledge about platform workers’ control and resistance, especially the role of algorithms, little is known about the institutional conditions that shape workers’ status and their relations with the platform. In other words, we know a lot about the micro-politics of the platform workers instead of the macro foundation of such labor politics. Second, few studies on platform workers have considered their internal variations. It is recognized that the differences among platform workers in different industries are huge (De Stefano 2016; Howcroft and Bergvall-Kreborn 2019), and we barely know the differences between platform workers in the same industry. A more varied and comparable study on platform workers further contributes to our understanding of this group.

## Labor regime of Chinese platform workers

To fill the research gaps, in theory, we go beyond the platform and link platform labor politics to state politics. The concept of the labor regime distinguishes the labor process from the political apparatuses of production that regulate and shape struggles in the workplace, which links state politics to factory politics (Burawoy 1985:87). Exploring the labor regime of specific workers means a detailed presentation of labor organization and the institutional forces that shape labor relations at the point of production. Through the case of food-delivery couriers in China, this article brings the concept of the labor regime back into the study of platform workers. It examines how institutional, technological, and managerial conditions regulate and shape those couriers’ working conditions and status. In the empirical study, we probe two different types of food-delivery platform couriers in China and examine their differences in working conditions and the forces behind them.

The theoretical agenda of the labor regime in Chinese labor politics traces back to Ching Kwan Lee’s formulation of disorganized despotism within reformed state-owned enterprises (SOEs). It analyzes how the reform measures in SOEs contribute to workers’ subordination to managerial force (Lee 1999; Cai 2002; Hurst 2004). Moreover, the arrangements shaping SOE workers’ status could also be found among migrant workers. Rich literature demonstrates how migrant workers’ labor power was exploited at the point of production, and their lawful rights were violated, implying an asymmetric power relation between labor and management (Chan, 2001; 2010; Lee, 2007).

The literature suggests two institutional arrangements regulating and shaping the despotic labor regime. The first aspect is the continual efforts of the state to channel labor relations into the juridical arena (Lee, 2002; 2007; Su and He, 2010; Gallagher, 2017; Estlund, 2017). However, the enforcement of legal regulations usually does not favor workers’ interests (Eli and Lee 2010; Pickles and Zhu 2015). The second aspect relates to the role of trade unions in shaping workers’ labor power. With a dual identity of a state agency and workers’ organization, the trade union often fails to represent workers’ interests, which leaves workers without formal organizational support (Chen 2003; 2009; Hui and Chan 2015).

Compared with the traditional manufacturing industry in China, the labor organization in the platform economy is undergoing a great transformation. However, the key institutional arrangements that regulate the labor field remain intact in the platform economy because of the continuity of Chinese labor relations institutions. To apply the conceptualization of the labor regime to food-delivery platform workers in China, we develop an analytic framework premised on the concept of the platform regime.

The platform regime is proposed here to replace the common usage of the labor regime. While the concept of the labor regime is a generalized articulation that links labor politics to state politics, the platform regime is specific to articulating the labor relations between platform workers and platforms. As evidenced by the literature, platforms and their algorithms play centralized roles in organizing the labor force and coordinating workers’ interests. Due to its centralized role, we believe it is a more suitable concept than a generalized labor regime to present platform workers’ status.

Similarly, Lei develops the concept of “platform architecture” to examine the technological, legal, and organizational aspects of control and management in the labor process to explain the variation in labor contention among food-delivery couriers (Lei 2021). Inspired by this concept, the platform regime cares about technological, legal, and organizational aspects revolving around the platform. However, while the platform architecture is developed to explain the collective labor contention, the platform regime is suited to the theoretical tradition of the labor regime. To encapsulate, the analytic agenda can be divided into two parts through the lens of the platform regime. First, we present the institutional context that each type of platform regime faces and explain how it lays the foundation for its despotic nature. Second, we show the labor organization process under each platform regime and analyze how workers are subject to the platform differently.

## Method and data

This study chose food-delivery platform workers in China as a case to elaborate the configuration of the platform regime for the following practical reasons. First, the food-delivery industry constitutes an important part of China’s platform economy. In 2019, the scale of the food-delivery industry in China reached 653.57 billion—39.3% higher than in 2018,^6^ attracting more than 7 million workers. Second, the status and working conditions of food-delivery couriers are the most contested and controversial in China’s platform economy because of their precarity. Studying this contested terrain can help us understand platform labor’s complex and multifaceted dynamics.

This study used qualitative research methods. The data mainly came from interviews of food-delivery couriers and participant observations at two service stations (*zhandian*) in two Chinese cities: Wuhan and Changsha. Other complementary data included online participant observations in a WeChat group consisting of 437 food-delivery workers in Wuhan and the analysis of a large number of relevant reports on food-delivery couriers and official documents of the food-delivery platform through websites such as “Baidu forum,” “Meituan forum,” and “Ele.me forum.”

In January 2020, the second author conducted ten in-depth interviews with eight food-delivery couriers and two food-delivery station supervisors in Wuhan. The second author participated in a large survey project on food-delivery couriers in Wuhan and had the chance to interview some of them. The interviewed couriers were male crowdsourcing couriers who were in their twenties. In July 2020, the third author interviewed 13 platform workers at a Changsha station. The third author entered the field through personal contacts and established rapport with some couriers who eventually became her interviewees. All the interviewees were male; most were married and approximately 30 years old. The interviews lasted between 30 minutes and an hour. The interview topics included their working experience, how their work was organized, the role of algorithms in the labor process, how these workers understood their labor process, and what strategies workers adopted to strive for autonomy. The second and third authors also conducted observations at two stations to observe the operation of the stations, including couriers’ morning assemblies and station supervisors’ coordination of order dispatches. To protect the privacy of the interviewees, all the informants use pseudonyms.

## Two types of platform regimes

Before examining the platform regimes among *Zhuansong* and *Zhongbao* couriers, it is necessary to describe the general working process for both types of food-delivery couriers. A food-delivery courier has to download the app, register to have an account, and prepare electric motors and food-delivery bags. The typical procedures for couriers to finish the delivery are as follows: (1) workers log into the platform app and wait for order requests; after getting order requests from the app, workers go to the restaurant addresses with the app’s navigation function. (2) Workers pick up food from the restaurants and confirm it on the app. The app will show workers the customer addresses and the dispatch time. (3) Workers reach customers within the time according to the route provided by the app navigation and confirm the deliveries on the app.

Despite the similar working process, the configuration of the platform regime is different between the *Zhongbao* and *Zhuansong* platform couriers. While platform despotism generally applies to food-delivery couriers, *Zhongbao* couriers are characterized by a regime termed individualized platform despotism, and *Zhuansong* couriers belong to bureaucratized platform despotism. We will present each platform regime with empirical data.

### Individualized platform despotism

Individualized platform despotism refers to a platform regime featured by an individualized labor process in which the platform app dominates, and *Zhongbao* couriers are solely and directly regulated and controlled by the algorithmic platform management. This despotism is manufactured by an institutional and legal ambiguity that fails to mediate the interests of the platform, restaurants, and *Zhongbao* couriers.

#### The institutional context of *Zhongbao* couriers

The app’s design makes *Zhongbao* workers independent contractors of the platform, who work independently and are responsible for themselves; the platform has no responsibilities for providing protection. *Zhongbao* workers are not permitted to sign a labor contract with any entities. For workers choosing to sign up as a *Zhongbao* type, the platform app designs a clickwrap agreement stipulating the food-delivery couriers as independent contractors rather than employees of the platform, and workers must accept the agreement to be a courier. Therefore, the platform can easily avoid its responsibilities when *Zhongbao* workers are involved in disputes. We extract some clauses from the M crowdsourcing platform to reflect *Zhongbao* couriers’ status and the conditions of their labor rights:

1. The platform delivery couriers are defined as persons with full civil capacities who accept and consent to all the rules and terms stipulated by the platform.
2. The crowdsourcing platform is an information platform that provides couriers with information on customer needs, the delivery fee per order, and confirmation of completed service. The couriers can autonomously choose their tasks.
3. The couriers are aware of and willing to take the risks of providing the food-delivery service. The crowding platform will not provide any type of explicit or implicit warranty.
4. The couriers must strictly follow the platform rules. The couriers will individually bear the corresponding responsibilities if they violate the rules and cause losses for the third parties or the platform.

Under the clickwrap agreement, *Zhongbao* couriers rely on the platform for providing service but are entitled to no protection or social welfare. It is also difficult for these couriers to confirm their employment status in China’s current labor jurisdiction. The Ministry of Labor and Social Security released “Issues Confirming Labor Relations” in 2005, implying that the labor relation can be confirmed only with evidence ranging from wage payment records and social insurance payment records to employee ID and attendance records. Employee status also means that an employee’s activities are part of an employer’s business and that an employee is under an employer’s command and control to complete the working tasks. However, this notification lags behind what these *Zhongbao* workers face, putting the confirmation of their employment status into question. According to the notification, there are evidential elements supporting either employee or independent contractor status for these *Zhongbao* couriers. On the one hand, they can decide when and where to work; at the same time, their labor organization is tightly guided and controlled by the technological system inside the platform. The existing legal system fails to classify *Zhongbao* workers as employees and endows them with the corresponding labor rights protection. This legal ambiguity of *Zhongbao* couriers makes them particularly vulnerable. For example, if a traffic accident happens, workers must adduce evidence to prove their labor relations with the platform by themselves during the litigation process, and the litigation process could be too costly to bear.

Regarding the collective organization of *Zhongbao* couriers, establishing a traditional workplace trade union does not apply to platform workers. Until now, the All-China Federation of Trade Unions has not created any trade union units among platform workers.

*Zhongbao* couriers’ vulnerability is further strengthened by the fact that the platform company can arbitrarily change the rules and terms of the platform without the agreement of the *Zhongbao* couriers. If the *Zhongbao* couriers do not agree to the changed rules and terms, the couriers can only terminate their accounts. Most *Zhongbao* couriers are migrant workers who are excluded from social welfare protection and establishing any collective organization to negotiate with the platform company.

#### Platform control in *Zhongbao* couriers’ labor process

*Zhongbao* couriers’ labor process is solely directed and controlled by the platform app. For *Zhongbao* couriers, the digitally-driven platform is a monopolized entity that dominates every detail of their labor organization (Moore et al. 2017), which means the platform becomes an algorithmized, nonnegotiable “employer” (Srnicek 2017:47).

Since the labor process builds upon the digitally enabled platform app, the app becomes the center of labor organization and service provision (Gandini 2019). The platform uses big data and algorithms to analyze couriers’ locations, restaurant locations, and customer locations to assign orders to the most suitable courier. With the algorithms, the platform directly assigns orders to the couriers. Couriers cannot determine their work content and must obey the algorithmic assignments of the platform. If the mandatory assignment is rejected, there is a heavy price for couriers to pay, such as a decreasing rate of obtaining orders and income loss.

*Zhongbao* couriers’ labor is highly affected by the “order dispatch index (*paidan zhishu* in Chinese),” which directly affects the quantity and quality of subsequent orders that couriers receive. This order dispatch index is affected by couriers’ behaviors, such as the number of rejected orders, the on-time delivery rate, the number of canceled orders, the number of complaints, and the couriers’ level. Every courier seems to have a digital document formed in the platform according to work performance. The platform uses the order dispatch index to “sort” couriers and establishes dispatch priority. The higher the dispatch index, the more likely couriers are to obtain better orders with a short distance and higher prices.

If *Zhongbao* couriers reject orders frequently, the dispatching system reduces their order amount, decreasing their income. Workers’ behaviors that do not conform to the interests of the platform are punished by the platform, which disciplines couriers to put more effort into the labor process. Although every courier has 2–4 chances to transfer orders a day, this must be taken by other couriers within 3 minutes. Even so, the more orders the couriers reject, the fewer orders they will receive in the future. If couriers want to deliver more orders and obtain higher income, they must maintain good performance.

To motivate *Zhongbao* couriers to work harder, the platform designs a differential payment system that gamifies workers’ labor process. The M platform initiates a weekly “Happy Running Rider” activity to encourage workers to work as long as possible. Workers’ wages are composed of two parts: the corresponding piece rate and a weekly bonus. The piece rate increases as the volume of workers’ orders increases. The bonus is awarded to those who reach specific valid online days in a week. For example, workers online for five days per week are rewarded with 160 yuan; the reward is 400 yuan for six days. It can be seen from the income structure that the food-delivery platform implements an accumulative piece-rate system. Under this system, income depends on the number of delivery orders couriers complete daily, which provides a strong incentive.

The study also finds that the platform encourages workers to exert more effort through multiple programs initiated by the platform, such as ranker rewards, severe weather subsidies, and peak-hour subsidies. For example, in the E platform, the food-delivery platform classifies couriers into ordinary knights, bronze knights, silver knights, golden knights, and diamond knights based on the scores couriers receive. Couriers receive one point for each completed order and an additional 0.5 point for each order during breakfast and dinner. The labor process is simulated as a game, with narratives and settings such as “killing monsters and upgrading.” The platform launches various activities on the app interface, like the game setting. Under this gamified setting, couriers can only choose to improve their working ability and dedicate more time to upgrade levels.

The platform can also use its algorithms to monitor the couriers’ labor process without any physical contact. Couriers’ moving statuses are visualized in the app and are tightly supervised by the platform app, including when they arrive at the restaurant and pick up the delivery, the route couriers take, and the time it takes to finish the delivery. The courier becomes a “number” and is calculated, programmed, and sorted by the platform algorithm. To gain an advantage in the competition, the couriers can only obey the system’s arrangements and follow the system’s dispatch instructions. This all-pervasive supervision of the platform is accompanied by asymmetrical information between the couriers and the platform (Rosenblat and Stark 2016). Without disclosing much information about the algorithmic operation, the platform maneuvers the supervision of couriers without generating much resistance from them.

The platform also encourages customers to evaluate couriers’ performance after the delivery is completed, which ultimately affects the assessment of couriers. The evaluation of couriers in the platform is relatively simple. For example, on the M platform, consumers have two options when evaluating the services: satisfied and dissatisfied. On the E platform, there are three options: very poor, average, and excellent. For both platforms, bad reviews mean fines. There are many reasons why couriers receive bad reviews, including low-quality food, a sprinkling of meals, wrong orders, poor service attitudes, and malicious or unintentional negative reviews by consumers, but the delivery timeout is the most common reason. Regardless of the reasons that couriers receive bad reviews, the consequences are borne by the couriers themselves, even though the platform estimates the delivery time that individual couriers have no method to control it. By transferring the evaluation of workers’ performance to customers, the platform quantifies the couriers’ services into data that are utilized by the platform as the basis for rewards, punishments, and restraints of the couriers’ behavior. The platform uses customer feedback to monitor, evaluate, and constrain workers, reinforcing the platform’s domination over the couriers.

With the aid of new technologies, the platform automates the control and management of *Zhongbao* couriers’ labor process through its digitalized and algorithmized order dispatch. To summarize, we examine how individualized platform despotism is configured among *Zhongbao* couriers in this section. The political apparatuses of legal ambiguity and the absence of social welfare protection and collective organization provide an institutional context for a despotic platform regime. Without managerial personnel, *Zhongbao* couriers’ labor process is individualized through the interaction between the couriers and the technological interface that is dominated by the platform.

### Bureaucratized platform despotism

There is a dualism between labor recruitment and organization because platforms cannot solely depend on *Zhongbao* workers to meet market needs. A more reliable and disposable labor force of *Zhuansong* couriers was created, characterized by the regime of bureaucratized platform despotism. The status of *Zhuansong* couriers is analogous to migrant workers who have been trapped in the dilemma of the nonenforcement of legal regulations (Gallagher 2017), which lays the foundation for a despotic labor regime. Unlike the *Zhongbao* workers solely dominated by platform algorithms, *Zhuansong* couriers’ labor process is subordinated to two authoritative entities: the bureaucratized service station and invisible algorithms.

#### The institutional context of *Zhuansong* couriers

China has promulgated multiple laws and regulations to protect workers’ labor rights. The Labor Contract Law, which came into force in 2008, stipulates that all employers must sign a work contract with their employees. A work contract is a springboard for employees to guarantee their basic labor rights of working time, wage payment, and social insurance programs, and state bureaus treat the labor contract as the most important evidence during labor mediation, arbitration, and litigation when dealing with labor disputes. Platforms usually transfer their juridical responsibilities to couriers’ labor rights by franchising their business to other franchisee companies. The franchisee is responsible for signing a labor contract with *Zhuansong* couriers, who are treated as employees. However, according to our informants, they barely realize the necessity to sign a contract with the service station. One informant working as a *Zhuansong* worker on the M platform told us that no one mentioned the contract issue when he was recruited and what he cared about was delivering more orders every month to increase his income. In her ethnographic study, Lei also mentioned that these *Zhuansong* workers usually have low expectations of social protection (Lei 2021). Therefore, there is low enforcement of labor law among these *Zhuansong* workers, which once again places workers in a precarious position vis-à-vis the platform or other managerial representatives.

For those *Zhuansong* workers who signed an agreement with the service station, it stipulates the length and content of the service, payment details, and the requirements of the working process. The following clauses are extracted from an agreement between a franchisee company and the couriers:

1. The couriers can arrange their working time themselves since the employment begins; the franchisee company is not obligated to pay social insurance fees or provide any economic compensation.
2. The couriers are responsible for delivering orders based on the labor needs of both sides. The couriers’ task is food delivery, and the working area is Wuhan and its suburbs.
3. The franchisee will pay the couriers daily only when the couriers finish the tasks and the franchisee finishes the couriers’ evaluation. The price for every delivery is 3–5 yuan, depending on the distance.
4. The couriers should hold a qualified license for motorcycling. If the couriers encounter traffic accidents without qualified licenses, all the consequences will be paid by the couriers.
5. The termination of the employment agreement will be dealt with according to stipulated procedures. Both sides can terminate the agreement at any time. When the couriers submit the application, the couriers can leave only when the franchisee consents and the couriers finish handing over the job.

It is obvious that the stipulated clauses in this employment agreement (rather than a labor contract) are despotic and unfavorable to the couriers. This despotic agreement barely provides any protection or social welfare to the couriers, while the couriers are under stringent regulations by the service stations. Furthermore, the couriers are excluded from state social welfare. Indeed, the service station only buys accident insurance for each courier, and the fee is deducted from the courier’s wage. At the same time, most *Zhuansong* couriers are also migrant workers who do not share social welfare protection as urban citizens.

#### Dual control of platform and service station in *Zhuansong* couriers’ labor process

The salient feature of *Zhuansong* couriers’ labor control is the workers’ double subordination to the managerial force of the service station and the platform algorithms. Compared with *Zhongbao* couriers, *Zhuansong* workers’ labor process is largely regulated by the service station. When franchising the business to the franchisee companies, the platform sets up key performance indicators (KPIs) for them to achieve, such as the number of total orders, on-time rates, and customer complaint rates. The platform can terminate the franchising contract if the franchisee companies fail to do so. To meet such KPIs, the franchisee and its service stations adopt several methods to control and manage *Zhuansong* workers.

First, the service station has a working schedule for the *Zhuansong* couriers, which means the workers could not decide when to log into the app and accept delivery orders. Based on our interview data, there are three working shifts for *Zhuansong* couriers in the station: the morning shift from 8 a.m. to 10:30 a.m., the noon shift from 10:30 a.m. to 4 p.m., and the night shift from 9 p.m. to 2 a.m. (midnight in winter). In addition to their respective shifts, all *Zhuansong* couriers must be online and accept orders during the noon and night peaks. Many *Zhuansong* couriers are aware that the flexibility and freedom of platform workers declared by the platform are fantasy. Even when they are not accepting orders, they are waiting outside the station to be dispatched by the platform.

Second, to improve *Zhuansong* couriers’ efficacy, the station supervisors adopt several disciplinary measures: (1) the couriers must have 27 days of attendance every month; otherwise, they lose the full attendance bonus. (2) The supervisor has a morning assembly for all *Zhuansong* couriers every day. The supervisor emphasizes the significance of teamwork and encourages couriers to work harder. The supervisor also blames couriers for misbehaving, such as late deliveries, a bad attitude toward customers, and breaking traffic rules.

Third, the station supervisor uses the technology provided by the platform to intervene in couriers’ labor process, including rearranging their order dispatch, mediating conflicts among couriers, and managing bad reviews from customers. The supervisors can monitor the delivery process through the technological system at the station. For example, the supervisor said that as the new courier may be late with the delivery, the supervisor can transfer the task to a more experienced courier to finish the delivery on time. When there are conflicts between couriers involving order dispatch (every courier wants orders with a short distance and a higher piece rate), the supervisor must mediate. The supervisor can delete bad reviews within a month from the background system when the quotas are within five; otherwise, the service station fines the couriers 30 yuan per bad review.

In addition to the organizational management of the station, *Zhuansong* workers also depend on the app and its algorithms to finish their food-delivery service, as we described among *Zhongbao* couriers. The algorithmic design functions to realize continuous time compression for capital, which makes couriers work in an unprecedentedly fast way (Li and Jiang 2020; Chen and Sun 2020). In the food-delivery platform’s business model, meeting customers’ timely needs is the primary task. The platform utilizes slogans such as “XXX takeout, fast delivery of everything” or “delivery on time, compensation for overtime.” The timeliness of time has become an important basis for the platform to produce high-quality services, and it is used as a criterion to discipline couriers. The platform has strict requirements on the timing of the food-delivery process, and punctuality has become an important evaluation criterion. The system automatically counts the time when the couriers receive the order to determine whether each order is late.

“Racing against time” has become a daily experience for couriers. Couriers do not often take one order each time but many orders at the same time. The platform will also send multiple orders to couriers to improve delivery efficiency and plan the optimal route for couriers. When the couriers take multiple orders at the same time, the delivery time becomes tight. Sometimes couriers even need to complete six orders within an hour, meaning the actual delivery time would be above 10 minutes instead of 30 minutes as customers see on the app. Therefore, any timeout will cause multiple orders to time out altogether. Based on a journalist report, the delivery time has been compressed during the last few years. In 2016, the maximum time for a three-kilometer delivery was one hour; in 2018, it was 38 minutes.^7^ The time gradually disappears on the platform. While time compression is proudly claimed by the platform as technological advancement, it is a tragedy for couriers. The continuous time compression sets the couriers into a trap of “race to bottom time”—the faster they run, the faster the algorithm operates, and the less time workers have for the delivery.

*Zhuansong* couriers’ double subordination to the bureaucratized service station and platform algorithms shows how these couriers directly rely on the service station and indirectly rely on the platform to participate in service work for their livelihood in the city. Due to the double subordination, the platform and its franchisee company can impose coercive modes of labor control. This double subordination has institutional roots: *Zhuansong* couriers’ labor rights are not well protected by the existing legal regulations, and there is no organizational foundation to support couriers’ collective bargaining with the platform company, the franchisee company, and its service stations. The combination of institutional apparatuses and their double subordination creates what we call bureaucratized platform despotism among these *Zhuansong* couriers.

## Conclusion and discussion

Labor process analysis has extensively been utilized to understand the dynamics of control and resistance among platform workers (Gandini 2019; Veen et al. 2020; Tassinari and Maccarrone 2020). Unfortunately, the concept of the labor regime that links state politics to labor politics is largely missing in the study of platform workers. This article aims to bring the labor regime back to the platform labor study and examines how the political apparatuses shape workers’ working conditions and status through the case of food-delivery couriers in contemporary China.

To elaborate on food-delivery couriers’ status and the institutional forces that shape it, we develop a concept of the platform regime that encompasses the legal, technological, and organizational aspects regulating the relations between the platform and workers. Furthermore, we compare two different types of food-delivery couriers and present two platform regimes that apply to dedicated delivery couriers and crowdsourcing couriers. Our arguments are twofold. First, despite its new mode of labor organization among food-delivery platform workers, the institutional ambiguity and nonenforcement of legal regulations lay the foundation for platform despotism in the food-delivery platform economy. Second, there are two types of platform regimes between *Zhongbao* and *Zhuansong* couriers due to different combinations of institutional, technological, and managerial elements. Individualized platform despotism applies to *Zhongbao* couriers who work individually and are regulated and guided only by the platform app and its algorithms. *Zhuansong* couriers belong to bureaucratized platform despotism, which emphasizes couriers’ double subordination to bureaucratized service stations and platform algorithms.

Due to data limitations, we do not elaborate on couriers’ labor politics. It would be sociologically interesting to explore how food-delivery couriers resist its control and how it differs from migrant workers in the manufacturing industry. The despotic platform regime among food-delivery couriers is expected to induce worker grievances and generate labor protests. Protests against food-delivery couriers in other countries have been recorded (Tassinari and Maccarrone 2020; Veen et al. 2020). In our data, while we did not encounter any collective labor protests, media coverage shows that the couriers could initiate strikes to defend their rights.^8^ However, until now, the scale, frequency, and coordinated mobilization have not been comparable with those of migrant manufacturing workers. Future studies could further examine how the labor protests of platform workers would be different from those in traditional industries.

The state plays a significant role in adjusting the labor relations between the platform and workers. Some local governments have already taken actions to regulate platforms’ misbehaviors and their franchisee companies. For example, multiple state bureaus in Nanjing collectively issued guidance on labor use in food-delivery couriers to protect couriers’ rights.^9^ Therefore, if the state continues to intervene to reduce workers’ dependence on the platform for labor power reproduction, set limits on platforms’ coercive managerial measures, and increase platform workers’ collective bargaining power, it is also possible that the despotic platform regimes could be transformed to hegemonic regimes.

## Data Availability

We based our study on qualitative data of participant observation and depth interview.

## Abbreviations

ICTs: Information communication technologies
SOEs: State-owned enterprises

## Footnotes

1 China National Information Center, “Annual Report on China’s Sharing Economy Development (2019)”, see http://www.sic.gov.cn/News/557/9904.htm, retrieved on 8 January 2021.

2 https://www.chyxx.com/industry/202106/956697.html, retrieved on 9 January 2022.

3 https://fengniao.ele.me and https://peisong.meituan.com, retrieved on 9 January 2022.

4 http://www.199it.com/archives/823693.html, retrieved on 3 March 2021.

5 https://pdf.dfcfw.com/pdf/H3_AP202004241378553199_1.pdf?1587845818000.pdf, retrieved on 3 March 2021.

6 The data were released by *Meituan Takeout*, a famous platform company in the food-delivery industry in China, see at https://www.sohu.com/a/409725269_99900352?_trans_=000014_bdss_dkwcdz12zn, retrieved on 10 January 2021.

7 https://new.qq.com/omn/20200918/20200918A0M0DN00.html, retrieved on 6 March 2021.

8 “Pessimism and Action 2020: An Inventory of Top Ten Workers’ Rights Protection Events”, see at https://mp.weixin.qq.com/s/aAae6IC6o0EIib5symqZZQ, retrieved on January 3, 2021.

9 See at http://rsj.nanjing.gov.cn/njsrlzyhshbzj/202104/t20210430_2902108.html, retrieved on June 7, 2022.
